# Does the physical environment matter? - A qualitative study of healthcare professionals’ experiences of newly built stroke units

**DOI:** 10.1080/17482631.2021.1917880

**Published:** 2021-07-09

**Authors:** Susanna Nordin, Anna Swall, Anna Anåker, Lena von Koch, Marie Elf

**Affiliations:** a School of Education, Health and Social Studies, Dalarna University, Falun, Sweden; b Department of Neurobiology, Care Sciences and Society, Karolinska Institutet, Stockholm, Sweden

**Keywords:** Evidence-based design, interviews, multi-professional, nursing, physical healthcare environment, rehabilitation, stroke

## Abstract

**Purpose**: Organized care in specialist stroke units is fundamental for achieving better outcomes for persons with stroke. Although the importance of the physical environment for health and well-being is well recognized, research regarding how environmental features can influence stroke care is limited. The aim was to elucidate healthcare professionals’ experiences of the physical environment in newly built stroke units with respect to stroke care.**Methods**: Healthcare professionals (n = 42) representing eight professions participated in semi-structured, face-to-face interviews. Qualitative content analysis was used.**Results**: The physical environment both facilitated and restricted the professionals’ ability to provide stroke care. Five categories were identified: “Working towards patient engagement in single rooms”, “Hampered rehabilitation in an environment not always adapted to patients’ difficulties”, “Addressing patients’ psychosocial needs in the environment”, “Ensuring patient safety by using the environment in accordance with individual needs”, and “Collaboration and task fulfilment—a challenge due to care unit design”.**Conclusions**: The healthcare professionals viewed the physical environment mainly in relation to stroke patients’ specific needs, and several environmental features were considered poorly adapted to meet these needs. The physical environment is essential to high-quality care; thus, the process of planning and designing stroke units should be based on existing evidence.

## Introduction

The physical environment plays an important role in promoting health and well-being for patients and providing supportive workplaces for staff in healthcare settings (Joseph et al., 2015; Ulrich et al., 2008). To achieve expected results in healthcare, design decisions should be based on the best available knowledge from research and practice together with evaluation of existing buildings, a process known as evidence-based design (Hamilton & Watkins, 2009; Ulrich et al., 2010). This approach may be of particular importance within stroke units, where patients suffer from serious conditions and are depending on high-quality care (Stroke Unit Trialists’ Collaboration, 2013). To ensure that hospital environments (e.g., stroke units) will support care activities and contribute to expected results such as improved physical and cognitive functions in patients, the user perspective is crucial. This article presents results from interviews with healthcare professionals in three newly built stroke units in Sweden.

### The physical environment and health outcomes

According to Harris et al. (2002), the physical environment involves architectural aspects (e.g., room size), ambient aspects (e.g., sound levels), and interior design aspects (e.g., furnishing). Several literature reviews have shown that well-designed physical care environments in hospitals can positively affect patients’ health, well-being and safety, together with staff effectiveness and comfort (Huisman et al., 2012; Ulrich et al., 2008). For example, contact with nature can relieve pain in patients with various medical conditions such as cardiovascular disease and cancer (Malenbaum et al., 2008) and reduce healthcare staff stress (Ulrich et al., 2006), while safe handrails and non-slippery flooring can reduce patients’ falls in acute-care facilities (Taylor & Hignett, 2016). Moreover, room size and placement of furniture and workstations can affect communication and teamwork in healthcare facilities in general as well as in hospital units (Gharaveis et al., 2017), while enhanced visibility has a positive impact on staff and patient safety (Pati et al., 2016; Zamani, 2019).

The physical environment is an important component of person-centred care and has considerable potential to help meet the needs of each individual and facilitate care processes (Edvardsson et al., 2008; McCormack et al., 2010). A large body of research has investigated environmental factors and person-centred care approaches within residential care for older people (Chaudhury et al., 2013; Hung et al., 2016; Morgan-Brown et al., 2013; Nordin et al., 2017; Zeisel, 2013). For instance, dining rooms with orientation cues, a homelike atmosphere and acceptable noise levels can improve the dining experience of persons with cognitive and functional disabilities (Chaudhury et al., 2013). One step towards person-centred care is single-room design (Henriksen et al., 2007), which has become a global trend in new healthcare buildings (Maben et al., 2016). However, there is an ongoing debate as to whether single-room or multi-bed rooms should be provided (Brambilla et al., 2019). The main arguments for single rooms have been the reduction of disease transmission (Ulrich et al., 2008) and protecting patient privacy (e.g., patients being able to control private conversations and social encounters) (Huisman et al., 2012; Maben et al., 2016). Among the arguments against single rooms are reduced staff monitoring, teamwork difficulties (Donetto et al., 2017; Maben et al., 2016) and patients being socially isolated (Anaker et al., 2019; Maben et al., 2016).

### Stroke unit care

Stroke occurs suddenly and unexpectedly, usually followed by a range of disabilities requiring long-term rehabilitation and resulting in major life changes for the individual (Crowfoot et al., 2018). Approximately one-third of stroke patients become permanently disabled with, for example, partial paralysis and impaired comprehension, memory and speech (World Health Organization, 2004) and are at risk of being affected by depression (Villa et al., 2018). Stroke units are geographically bounded areas in hospitals exclusively for stroke patients (Ringelstein et al., 2013), and organized care in stroke units is fundamental for achieving better outcomes for persons with stroke (Stroke Unit Trialists’ Collaboration, 2013). Essential stroke care activities involve the detection and management of complications together with early mobilization based on the patients’ individual needs (Powers et al., 2019; Ringelstein et al., 2013; Stroke Foundation, 2017). A key component of effective stroke care is the provision of holistic and comprehensive care covering a range of different interventions performed by multi-professional teams with expertise in nursing, medicine, and rehabilitation (Langhorne & Pollock, 2002; Miller et al., 2010). Such teamwork involves careful choreography to provide optimal patient care, including communication and collaboration among healthcare professionals (Taylor et al., 2015) and emphasizing the participation of patients and their relatives (Kirkevold, 2010; Miller et al., 2010).

### Environmental research in stroke care

In recent years, there has been an emerging interest in stroke care environments, and observational studies have investigated how the physical environment can support stroke care and rehabilitation (Anaker et al., 2018; Shannon et al., 2018), including enriched environments (Janssen et al., 2014; Rosbergen et al., 2019; White et al., 2014). For instance, patient access to social meeting places (e.g., day rooms) and opportunities for activities via computers, books and games can have a positive impact on well-being and promote rehabilitation (Janssen et al., 2014). The occurrence of patient activity in these facilities has been observed, and several studies found that patients in both acute and rehabilitation stroke facilities were inactive and alone for a large part of the day (Anaker et al., 2017; Astrand et al., 2016; Bernhardt et al., 2004), which is likely to negatively affect recovery. However, research on staff perceptions of the physical environment in stroke units is still limited. Seneviratne et al. (2009) are among the few to have taken a staff perspective, and they found that nursing staffs’ opportunities to move around, to use equipment and to transfer patients together with documenting care and interacting with stroke team members were hampered due to limited space.

### Evidence-based design

As a result of the awareness of the environmental impact on people’s health and well-being, evidence-based design (EBD) has evolved. EBD is based on evidence from various disciplines and involves representatives from healthcare, architecture and building construction (Elf et al., 2015). It is a critical process in which current evidence and experiences from existing environments are used (Stankos & Schwarz, 2007) and the needs of the users are identified (Hamilton & Watkins, 2009). Nevertheless, such evidence is not always transferred into building standards and regulations (Vischer, 2008), and there is limited feedback on how the physical environment works for its users, e.g., staff and patients, after a new facility has been put into use (Leaman et al., 2010; Vischer, 2008). This limited evidence also applies to stroke units, where more knowledge is required regarding how environmental features influence stroke care. Hence, it is of great value to consider the views of the healthcare professionals working in a highly specialized care environment and who are responsible for providing vital care to people affected by serious conditions such as stroke.

### Aim

The aim of the present study was to elucidate healthcare professionals’ experiences of the physical environment in newly built stroke units with respect to stroke care.

## Methods

### Design

To capture the healthcare professionals’ experiences of the physical environment, the study employed a qualitative research design using semi-structured interviews. Qualitative content analysis with an inductive approach was considered appropriate to allow new insights to emerge from the data (Elo & Kyngäs, 2008). The study was reported in accordance with the consolidated criteria for reporting qualitative research (COREQ) checklist (Tong et al., 2007).

### Context and participation

The study was guided by the assumption that new facilities would be designed to support stroke care in accordance with current knowledge (e.g., early rehabilitation provided by a multi-professional team to address the needs of the individual). The criterion for inclusion was that the unit should have been subject to a fundamental change in the design of the physical environment. An example of such a change is the patient room design, where multi-bed rooms had been replaced with single rooms and where patients have access to their own bathroom. Another criterion was that the units were built or inaugurated after the year 2009, when the Swedish stroke guidelines were published.

The stroke units were identified through the Swedish Health Care Facilities Network, a forum for organizations and professionals working with healthcare facilities. The included stroke units were located at three hospitals in different counties in Sweden. All of the included stroke units provided a combination of acute care and rehabilitation. In two of the units, single-room accommodations predominated, whereas one unit had a mix of single rooms and multi-bed rooms, with the majority being single rooms. All the units had therapy areas located on the same floor as the unit and access to venues such as patient lounges. Staff workspaces consisted of lockable rooms, and two of the units also had open working desks in the corridors near the patients’ rooms. More details on the stroke units’ environmental characteristics are presented in Table I. All healthcare professionals on the selected stroke units who had worked for at least one year were eligible for inclusion.

**Table I.** Environmental characteristics of stroke units

Stroke unit ID/Type of hospital	Type of stroke care	Construction/ renovation	Patient room types/Number of beds	Patient room facilities	Access to activity/therapy area	Day areas	Staff workplaces
Unit 1/University hospital	Acute stroke care and strokerehabilitation	Newly built	A mix of single rooms and multi-bed rooms with an overbalance towards single rooms. In total 23 beds.	Windows to the outside. Bathrooms within all rooms.	Located at the same floor as the patient rooms	Patient lounge/dining room located at the unit entrance with windows facing the outside. A glazed balcony adjacent to the lounge.	Several workplaces for staff, and open workplaces/desks located in the corridors. Designated rooms for team conferences, one located inside the unit, and one located close to the unit.
Unit 2/Regional hospital	Acute stroke care and stroke rehabilitation	Completely redesigned and renovated	Mainly single rooms. Access to two rooms with three beds in each room reserved for patients in need of medical monitoring. In total 22 beds.	Windows to the outside. Bathrooms within all rooms.	Located at the same floor as the patient rooms	Patient lounge/dining room located in the middle of the unit with a large open area in direction to the corridor. No windows, only artificial light.	Several workplaces for staff, and open workplaces/desks located adjacent to staff workplaces. No designated rooms for team conferences.
Unit 3/University hospital	Acute stroke care and stroke rehabilitation	Completely redesigned and renovated	Mainly single rooms. Access to one room with three beds reserved for patients in need of medical monitoring. In total 22 beds.	Windows to the outside. Bathrooms within all rooms.	Located at the same floor as the patient rooms	Patient lounge/dining room located at the end of one of the corridors. Windows facing the outside.	Several workplaces for staff. Designated rooms for team conferences.

### Data collection

For all three stroke units, data collection occurred over a period of 31 months in total. The data were collected by well-trained research assistants with an MSc degree and took place at the stroke units. Prior to data collection, the research assistants visited the stroke units to provide information about the study and to become acquainted with the environments. Face-to-face interviews were conducted with participants on one occasion using a semi-structured interview guide developed by the research team. The interviews lasted between 10 and 30 minutes, with the majority being 30 minutes. All interviews were audiotaped and transcribed verbatim. The participants were encouraged to talk freely about the topic, and each interview session started with an open-ended question: *Could you please tell me about your experience of the physical environment in the unit?* This question was supplemented by questions regarding specific features of the environment, e.g., *Could you please tell me your experience with the lighting in the unit?* Questions on how environmental factors could relate to care were also posed, e.g., *How do you think the environment affects opportunities for early mobilization?* To obtain a deeper understanding or clarify the meaning of the responses, follow-up questions were asked continuously during the interviews. Although similar questions were asked to the study participants, their individual experiences of the physical environment were in focus during the interviews.

### Ethical considerations

The study was approved by the Ethics Review Authority in Sweden (Ref No. 2012/199).

The World Medical Association (WMA) Declaration of Helsinki—ethical principles for medical research involving human subjects were followed (World Medical Association, 2013). Before informed consent from the participants was obtained, verbal and written information was provided on the study purpose and on what participation would entail. This information was repeated verbally to the participants in conjunction with the interview together with the information that they were free to withdraw from the study at any time without declaring any further reason. Verbal informed consent was obtained from all participants prior to the interviews, and the provision of consent was documented. The interview material was handled confidentially.

### Data analysis

To transform the interview text into findings and provide knowledge relevant to our study topic, content analysis was considered appropriate (Krippendorff, 2004). Content analysis involves both a quantitative and a qualitative methodology. Within quantitative research, it has a long history with roots in logical positivism (Berelson, 1952), while qualitative content analysis can be linked to the hermeneutic tradition originating in the interpretation of ancient and biblical texts (Grondin, 1995). In recent decades, qualitative content analysis has been a frequently used method focusing on interpreting human experiences. In this study, a qualitative content analysis method was applied. The interview material was analysed on a manifest level and conducted in accordance with several steps described by Graneheim and Lundman (2004). The first step was to obtain a sense of the whole, and the transcribed interviews were read and reviewed several times. In the next step, meaning units were extracted from the interview transcripts. The meaning units consisted of phrases and sentences relevant to the aim and related to each other through their content and context. Thereafter, the meaning units were condensed into shorter text and labelled with codes that concisely described their content. All codes were compared for similarities and differences before abstracting them into preliminary sub-categories and categories.

According to Krippendorff (2004), categories must be rooted in the data from which they originate, and no data relevant to the study purpose can be excluded because of a lack of a suitable category. Additionally, data should not fall between two categories or fit into more than one category. Two of the authors (SN and ME) carried out parts of the analysis together and discussed issues and uncertainties that emerged. Then, the first author took the lead of the analysis, which was characterized by an iterative process with a continuous movement back and forth between the original text, codes and categories. The preliminary categorization was revised after several discussions among all of the authors, who have experience both in conducting research in this field and in qualitative content analysis. Examples of the analysis process are displayed in Table II.

**Table II.** Examples of the analysis process

**Meaning units**	**Condensed meaning units**	**Codes**	**Subcategories**	**Categories**
I experience it as positive that the patients have their own rooms so that I am coming in to the patient and that both assessment and treatment are taking place in the patient’s room a bit on the patient’s terms	As the patients have their own room, assessment and treatment can be on the patient’s terms	More focus on the patient in single rooms	Caring for the patient on her or his own terms is facilitated in single rooms	Working towards patient engagement in single rooms
What I think can be difficult, is this rehab conference in this oblong room when everyone is sitting with their heads twisted, instead of everyone sitting around a table where we all can look each other in the eyes and so we are sitting and looking askew up at a projected computer, and that is not optimal in teamwork	Not optimal in teamwork when everyone is sitting with their heads twisted during the rehab conference instead of sitting around a table looking at each other	Difficult to see each other during conference	Varying prerequisites for team dialogues and documentation in work areas	Collaboration and task fulfilment – a challenge due to care unit design

## Results

In total, 42 healthcare professionals were included (assistant nurses, n = 10; registered nurses, n = 7; physiotherapists, n = 8; physicians, n = 6; managers, n = 3; occupational therapists, n = 4; speech and language therapists, n = 3; welfare officer, n = 1). The findings showed that the healthcare professionals at the three stroke units experienced the physical environment as both facilitating and restricting their ability to provide stroke care. The analysis resulted in the identification of five categories with underlying subcategories (Table III). The categories are presented with quotations from the interview text and represent participants from all stroke units.

**Table III.** Subcategories and categories

Subcategories	Categories
Caring for the patient on her or his own terms is facilitated in single rooms Family involvement in care is promoted in single rooms Privacy is facilitated in single rooms but restricted in multi-bed rooms	Working towards patient engagement in single rooms
Transfers and exercises require functional design and adequate space Transfers and exercises are restricted by non-purposeful furniture Environmental distractions as barrier and support	Hampered rehabilitation in an environment not always adapted to patients’ difficulties
Social interactions are restricted by inadequate communal spaces Rest and recovery are hindered by high sound levels Positive distraction is promoted by access to nature Opportunity to withdraw versus risk of loneliness in single rooms	Addressing patients’ psychosocial needs in the environment
The patient’s form of disability guides the use of aids and furniture Patients with a risk of falling are placed to enhance staff supervision Monitoring of patients is promoted by a central location and proximity to spaces	Ensuring patient safety by using the environment in accordance with individual needs
Availability at the expense of being interrupted in workplaces with open designs Limited privacy in workplaces Varying prerequisites for team dialogues and documentation in work areas	Collaboration and task fulfilment—a challenge due to care unit design

### Working towards patient engagement in single rooms

Healthcare professionals reported that caring for the patient on her or his own terms was facilitated in single rooms. When medical rounds, assessments and dialogues took place in single rooms, the healthcare professionals could engage the individual patient to a greater extent than in multi-bed rooms as it was easier to adjust to individual patients’ needs and share information. An occupational therapist described the advantages of single-room occupancy:

*Our assessments are very much based on conversations, which means that single rooms are great. We can sit down in peace and quiet; the patient can concentrate on what to do* (P6, Unit 2).

The involvement of family members and relatives in the care was promoted in single rooms, and the healthcare professionals described their opportunities to communicate with the family and exchange information during different stages of care. The results also showed that patient privacy and confidentiality were seen as important values and provided a basis for patient participation in care. These values were regarded to be facilitated in single rooms as explained by a nurse:

*One can actually talk about everything, and the patients can be asked about everything, and no one else can hear* (P7, Unit 2).

The healthcare professionals expressed concerns that multi-bed rooms restricted patient privacy and confidentiality as other people could overhear patient-provider conversations. A physio-therapist described her thoughts around patient examinations taking place in multi-bed rooms:

*Sometimes it doesn’t feel very good because there can be several patients in the room—it can be relatives, it can be other staff—and given the patient privacy, I’m not really happy with it* (P7, Unit 1).

When other people were present in the patient’s room, the healthcare professionals strived to protect the patients by speaking quietly and not carrying out more thorough examinations.

### Hampered rehabilitation in an environment not always adapted to patients’ difficulties

According to the healthcare professionals, patient transfers, exercises and other daily life activities required functional design and adequate space. For example, contrasting colours could help patients to navigate the environment, whereas monotonous features such as corridors that looked the same could be confusing. Activities that involved several staff members or required aids and technical equipment were regarded as particularly dependent on adequate space. A physiotherapist described a situation involving a patient with neglect:

*She [the patient] has left-sided neglect and needs to orient herself to the left and get up in that direction, but then it is crowded and cumbersome because there is too little space and too many other things around. So, it is not natural for the staff to help her get up in that direction* (P15, Unit 1).

The healthcare professionals described that many daily life activities took place in the patients’ bathrooms. Although proximity to hygiene areas was seen as highly beneficial, the bathroom was generally considered poorly adapted for stroke patients, with inadequate room for shower stools and wheelchairs, toilet doors opening in the wrong direction, insufficiently sloped floors and misplaced washbasins. Moreover, transfers and exercises in the patients’ rooms were restricted by the presence of non-purposeful furniture. These issues were described by a speech and language therapist and a physiotherapist respectively:

*Then, the patients do not reach the tables at all times. Because some wheelchairs are very wide, they [the patients] cannot reach the training tables in their rooms (P10, Unit 3).*

*The tables in the room are small, cute coffee tables that a stroke patient has no use for whatsoever. Instead, they need tables that they can sit at, exercise at and perform self-directed exercises and so on. These don’t exist* (P19, Unit 1).

The healthcare professionals also reported that distractions in the physical environment were seen as both a barrier and a support for the patients. For some patients, cramped and messy spaces could be stressful, while for more independent patients, these issues could offer valuable challenges such as taking a walk in the corridor and dealing with environmental obstacles.

### Addressing patients’ psychosocial needs in the environment

The healthcare professionals highlighted the patients’ psychosocial needs in the stroke unit in terms of both socializing with others and having access to privacy and rest. For example, concerns were raised that social interactions could be restricted by inadequate communal spaces for patients and visitors. An occupational therapist expressed her thoughts around this issue:

*One would have liked to have a day room that accommodates all patients, maybe a day room that encourages social interaction in another way* (P6, Unit 2).

The healthcare professionals also thought that stroke patients’ need for rest and recovery could be hindered by high sound levels. The corridors seemed to be particularly noisy as there was a consistent flow of people and noise from alarms, and concerns were raised that the patients had to cope with crowded surroundings despite their health condition after the stroke. A welfare officer described the following:

*It’s just that it’s a lot of people of course. It is a large unit, and it is a lot of staff; it is a rather busy environment sometimes, I think, for our stroke patients, who will suffer from brain fatigue* (P20, Unit 1).

For patients undergoing long-stay hospital care, the healthcare professionals described that positive distraction was promoted by views of the outside and contact with nature. A physician expressed the value of having access to the outdoors:

*As many stroke patients have possible occurrence of depression and homesickness, it is beneficial to go out and take a walk if the outdoor environment allows* (P4, Unit 3).

The healthcare professionals emphasized patients’ opportunities to withdraw but also raised concerns of loneliness in single rooms. They perceived that single rooms offered a quieter environment and were an alternative to the dining room for some patients. At the same time, the patients were perceived to spend considerable time in their rooms with the risk of becoming lonely.

### Results

There is inconsistency in terms of line spacing before quotes. For instance at page 8 there is space before the quotation “Then, the patients do not reach…Further down the page, there is no line spacing before the quotation “One would have liked to have a day…”

In other articles from the journal, there is often space before the quotations, which makes it very clear and easy to read. Also, in some places there seem to be different distances between headings and text.

### Ensuring patient safety by using the environment in accordance with individual needs

According to the healthcare professionals, the individual patient’s form of disability guided the use of the environment through aids and furniture. For example, impaired sensation was described as a source of safety risk that needed to be taken into account when using furniture, as noted by a speech and language therapist:

*These are height-adjustable tables, so if the patients push the button themselves, they can lower the table on a paralysed hand that is unable to feel anything* (P3, Unit 3).

Another example was cognitively affected patients who did not always understand their limitations, and to reduce the risk of injury, individual adjustments had to be made when using aids and furniture. The healthcare professionals also reported that patients with a risk of falling were placed in spaces that enhanced staff supervision. A nursing assistant explained this as follows:

*Usually, there are relatives who may be checking on the patients, but at night, we usually, if there is a really worried patient that we are not able to monitor, we can place that person in the corridor, although we are not supposed to …* (P2, Unit 3).

Centrally located workstations and patient rooms located near staff areas were described by the healthcare professionals to facilitate their ability to observe and hear patients calling for help or assistance. Nurses, in particular, required close proximity to patients as well as access to patient information and aids, since long distances between the nurse and the patient were regarded to jeopardize patient safety. Single rooms were regarded to be more problematic in terms of patient monitoring, and some healthcare professionals raised concerns about safety risks and thought that it was easier to be aware of what was happening in multi-bed rooms.

### Collaboration and task fulfilment—a challenge due to care unit design

The healthcare professionals expressed that in workplaces with open designs, their availability was achieved at the expense of being interrupted as these designs created a thoroughfare for many people. This meant that staff were highly exposed to the goings on in the unit. A nurse described both the advantages and disadvantages:

*That [open-designed desks] invites patients and relatives to make contact easily, and they dare to ask without feeling restricted … they feel that we are very available. The downside is that there are very many others standing there all the time … It is not a very tranquil environment to sit and concentrate in* (P17, Unit 1).

The limited opportunities for patient privacy in workplaces were reported by the healthcare professionals. Only at workstations with lockable doors could privacy be maintained, but it was highly limited at open-designed workstations. A nurse expressed her concerns about not being able to maintain patient privacy:

*Very poor [privacy]. As we have the rooms right behind us, the phone rings there, I might call and give a report on a patient, papers are often on the desk, even though I myself try to put them upside down so that you do not, so that it is not shown, from the sides of the computer; we do not have any privacy protection in that module, so there, one can see from the side. No, poor I would say* (P11, Unit 1).

The healthcare professionals raised the need for communicating with colleagues within the unit, which was both facilitated and restricted due to environmental design. A physician described accessibility to healthcare staff:

*Then, I think there are no long distances between us if I need to talk to someone. So, I usually find them … And just like the physiotherapists are sitting here too, and the occupational therapists, so if you have a question, it is easy just to drop by* (P2, Unit 2).

Moreover, the healthcare professionals described that team dialogues and documenting care were facilitated when there was adequate space, light and ventilation in the work areas, whereas noisy and crowded environments led to disruptions. Further, staff had to move between different rooms to get access to a computer, which resulted in work interruptions. The nurses in particular seemed to be frequently interrupted. One reason given was that the physicians’ seats were prioritized, and the nurses had to find another space for themselves.

## Discussion

This study explored healthcare professionals’ experiences of the physical environment in three newly built stroke units with respect to stroke care. It became apparent that the healthcare professionals mainly viewed the environment in relation to stroke patients’ needs and disabilities such as reduced spatial and bodily awareness, neglect, weakness, fatigue and depressive mood. Hence, a consistent focus in the interviews was on how environmental features could facilitate and restrict stroke care activities in relation to the physical, psychological, cognitive and social needs of those with these disabilities.

The patient’s room was a venue for care interventions such as medical rounds, assessments and dialogues, and our results showed that patient engagement was facilitated in single rooms.

Healthcare professionals could involve the patients and their relatives in the care as the needs and privacy of the individual patient were emphasized in single rooms. These findings are similar to those of previous studies showing that single rooms supported communication between staff, patients and family (Chaudhury et al., 2009; Maben et al., 2016); facilitated clinical examinations and conversations (Chaudhury et al., 2009; Persson & Määttä, 2012); and enhanced privacy (Maben et al., 2016). After a stroke, the ability to concentrate can be affected, and for many people with stroke, it becomes difficult to follow conversations, complete activities and learn new things (Miller et al., 2010). Thus, it is reasonable to assume that people with stroke are particularly sensitive to the surrounding environment during care interventions. A recent study conducted in stroke units showed that single rooms could offer a quieter environment than multi-bed rooms; the latter were found to negatively affect care due to noise and the fact that several activities were often being conducted simultaneously (Anaker et al., 2018). Additionally, more difficult conversations between staff and stroke patients were found to be restricted due to the lack of single rooms for patients following an acute stroke (O’Halloran et al., 2012).

However, our findings also indicated negative aspects of single rooms as the healthcare professionals raised concerns about loneliness and that patients spent considerable time in their rooms. These findings corroborate studies showing that hospital inpatients spend most of their day alone and inactive (Anaker et al., 2017; Kuys et al., 2011; Shannon et al., 2018), even when rehabilitation is the main goal of hospital admission (Janssen et al., 2014; Shannon et al., 2018). Although there could be several reasons for this, such as organizational factors (e.g., ward culture and staffing) or factors related to the patient (e.g., fatigue and pain), recent research in neurological settings stressed that environmental factors need to be considered (Blennerhassett et al., 2018; Shannon et al., 2018). Since early rehabilitation including mobilization and daily life activities is crucial in stroke care, the physical environment should be designed to encourage such activities. This could be applied not only in communal spaces but also in the patients’ rooms, especially when the prevailing trend is single-room occupancy. In addition, the design of the environment has the potential to enable activities based on individual needs, conditions and preferences. For instance, by providing the patient room with books, puzzles and other items of interest to the individual patient (White et al., 2014) together with a variety of furnishings (e.g., window seats), activities can be promoted (Shannon et al., 2018).

Another result was that patient monitoring was limited in single rooms, creating risks for patient safety, and the healthcare professionals expressed that it was easier to observe what was happening in multi-bed rooms. A possible reason may be that staff have fewer errands in single rooms and therefore are not aware of what is happening, whereas multi-bed rooms allow roommates to keep track of other patients’ conditions and call for help. Previous research has also shown that single-room design could reduce staff visibility and monitoring of patients, thus jeopardizing patient safety (Donetto et al., 2017; Maben et al., 2016). For instance, Donetto et al. (2017) found that poor opportunities for staff to keep an eye on several patients at the same time was a source of anxiety and dissatisfaction. Instead, in environments that enabled people to see and hear what was happening, patients could help each other and support care by warning healthcare staff about situations requiring immediate attention. Further, a recent study showed that the doors to patient rooms in stroke units were closed for most of the day, which may have contributed to patients being invisible to staff (Anaker et al., 2018). To enable assessments and observations of patients in single rooms, new work routines may be required such as more frequent visits to patients. However, more knowledge is needed about how care should be organized to meet patients’ needs and ensure that staff can perform care that is safe, efficient and person centred in facilities with single-room design.

According to our study, the healthcare professionals perceived the unit layout to be problematic for stroke patients who had to cope with crowded surroundings despite their need for fewer stimuli and a shielded environment. This finding is in line with the study by O’Halloran et al. (2012), who found that background noise and visual distractions in acute stroke units posed communication difficulties for patients with physical and cognitive impairments. Our results also indicated that the stroke units did not offer pleasant social areas for patients and visitors. Previous studies have shown that neurological patients are rarely seen in communal areas (Anaker et al., 2018; Shannon et al., 2018). Due to the varying and complex sequelae after a stroke, such as psychological and emotional complications, it is of utmost importance to offer a healthcare environment that supports patients in several ways and contributes to their well-being. Depression and anxiety are common post-stroke (Chun et al., 2018; Towfighi et al., 2017); they have a significant impact on recovery (Hackett et al., 2005) and are associated with lower engagement in stroke care activities (Villa et al., 2018). It is well recognized that stimulating activities such as engaging in hobbies can promote well-being for patients with stroke (Janssen et al., 2014), and enriched physical environments for acute stroke care have been shown to significantly increase patient activity and make patients spend more time outside their rooms (Rosbergen et al., 2019, 2017).

Further, social relationships play an important role for well-being for people with disabilities (Tough et al., 2017). Opportunities for social activities and family visits are therefore essential, and some researchers suggest introducing more variation in the design of the physical environment such as via small communal areas placed around the stroke unit for meetings and social interactions (Anaker et al., 2018; Rosbergen et al., 2019). However, inviting areas for communal interaction in the ward might not be enough. Shannon et al. (2018) found that virtually no patient activity took place in communal areas of the hospital care units despite new environmental designs such as a lounge and a therapy room. Several reasons were suggested. For instance, the absence of wayfinding cues and references in communal areas might restrict patients’ access to such areas, while well-designed patient room furnishings can promote activities within the patients’ rooms.

Importantly, the healthcare professionals in our study perceived the interior furniture to be poorly adapted to stroke patients and stated that it could restrict patient activities. This finding is in line with the study by Kuys et al. (2011), which showed that a majority of chairs in medical wards were not appropriate for patients to use while out of bed, with implications for their functioning. Moreover, our study findings indicated that daily care activities could be problematic due to poorly designed environmental features in the hygiene areas, and the interviews revealed that the environment was not adapted to meet the needs of patients affected by a range of disabilities post-stroke. Previous research has emphasized the importance of adequate furniture and relevant equipment for supporting the patients and enabling staff opportunities to perform safe and efficient care (Chaudhury et al., 2009). In current stroke guidelines, there is an emphasis on the importance of maintaining and regaining functioning and helping patients relearn basic skills for daily life activities such as washing, using the toilet and getting dressed (Boulanger et al., 2018; Miller et al., 2010; Ringelstein et al., 2013). These basic activities are important for the patient to be able to return home, and there appears to be potential for improvement in terms of environmental support in patient rooms and hygiene areas.

Our study findings showed that the healthcare professionals felt that the unit design influenced collaboration and task fulfilment in their daily work. Open-design workspaces promoted valuable contact and communication but also led to frequent interruptions of work. Inadequate space and ambient issues such as high noise levels and poor air quality were also regarded to have a negative effect on their work. Previous research has demonstrated the link between unit layout and the work of staff. For instance, teamwork and communication can be strengthened by co-location of different professionals on the team (Clarke, 2010), by open-plan environments enabling informal exchange of information between staff (Donetto et al., 2017), and by adequate spaces for team members to collaborate (Gharaveis et al., 2017). Teamwork and communication, including face-to-face communication, are crucial factors in delivering high-quality care (Kilner & Sheppard, 2010), not least within stroke care, where the multi-professional team plays a key role (Langhorne & Pollock, 2002; Miller et al., 2010). In our study, the physical environment did not always seem to support the work of staff. Although open workplaces can facilitate communication, they may also be problematic for staff to complete their tasks and maintain patient privacy. Thus, the stroke care environment should be designed with a variety of spaces, including meeting rooms and workplaces supporting communication and enabling staff to work together as a team, and to perform tasks such as documentation without being disturbed.

## Methodological considerations

A text always contains several meanings and involves different degrees of interpretation (Graneheim & Lundman, 2004; Krippendorff, 2013). To ensure trustworthiness in qualitative research, several strategies are therefore required. According to Elo et al. (2014), data collection, analysis and reporting of the results need to go hand in hand to ensure trustworthiness. In the present study, trustworthiness is discussed below by using the criteria for credibility, transferability, dependability and confirmability (Lincoln & Guba, 1985; Polit & Beck, 2012).

An essential aspect of credibility is the selection of an appropriate data collection method and representative participants to fulfil the aim of the study (Elo et al., 2014). A qualitative study using semi-structured interviews was considered suitable for capturing and understanding the experiences of the healthcare professionals. The study participants were registered nurses, assistant nurses, physiotherapists, physicians, managers, occupational therapists, speech and language therapists, and a welfare officer. They represent the multi-professional team whose work is of crucial importance within a stroke unit, and the ability to illuminate a variety of issues was regarded to be a key strength of the present study. However, it is important to keep in mind that the results reflect the perspectives of one user group, which may differ from other users of the stroke environment, such as patients and relatives. Another strength was the opportunity to interview staff from three hospitals in three different counties, as data on the same topic under investigation in multiple sites were regarded as enhancing the credibility. A study limitation was that some of the interviews were short (10 minutes). One reason may be that these participants had difficulties in expressing their thoughts around the physical environment. It can be assumed that staff with different experiences and professions have varying demands of the physical environment and thus describe this differently. Another assumption is that the environment is not considered as an integral part of care and thus find it more difficult to reflect upon. Further, since the interviews were conducted at the stroke units, it cannot be ignored that the participants may have felt compelled to return to their tasks. However, the majority of the interviews were approximately 30 minutes. Overall, the material was considered to provide rich information.

Further, credibility can be enhanced during the data analysis process by selecting the most suitable meaning units and ensuring that all data are covered by categories or themes. Although information could have been overlooked during the analysis process, this risk was considered low as the researchers repeatedly returned to the original text to ensure that no relevant data were left out and that interpretations were grounded in the data. Dependability can be enhanced by reporting how the findings were reached. In the present study, each stage of the research was described together with an example of the analysis process. Dependability also involves stability over time and under different conditions, and since our data collection took place during a period of 31 months in total, there was a risk of inconsistency when collecting data. However, the use of the same semi-structured interview guide in all interviews in the different settings was perceived to have strengthened dependability.

Transferability refers to the possibilities of applying the study results to other contexts. Although it can be problematic to generalize findings from qualitative research studies, the results from the present study might be transferable to similar settings (stroke units providing acute care and rehabilitation). However, this possibility has to be evaluated by the reader. Hence, the included stroke units were described in detail in the methods section. A limitation is the incomplete information on the study sample. For instance, data on age, gender and length of work experience would have contributed to a more comprehensive picture of the characteristics of the participants. Finally, confirmability refers to objectivity and that the study results represent the information obtained from the participants. Although the first author took the lead in the analysis, all of the authors repeatedly discussed and reflected upon the analysis process and development of sub-categories and categories. Furthermore, quotations from participants were carefully chosen and enhanced the understanding of the study results.

## Conclusions and implications

The present study adds to a growing body of literature on the meaningful role played by the physical environment in providing high-quality care. The healthcare professionals at the three stroke units experienced the physical environment as both facilitating and restricting care, but several features of the environment were considered to be poorly adapted to the specific needs of people with stroke. As all the included stroke units were newly designed, this finding is noteworthy. However, the healthcare professionals did their utmost to focus on the patients’ needs by adapting to the environment and the prevailing circumstances. Environmental features such as unit layout and room size are determined at the beginning of a design process. Therefore, we need to learn more about how such features can facilitate or restrict care and rehabilitation and use this knowledge in the planning of new stroke care buildings. Additionally, it is important to remember that the design of the physical environment is part of a complex system consisting of organizational policies, care models and daily routines as well as characteristics of the users of the environment. According to a global trend, single-room design is standard. Thus, care routines need to be adapted with, for example, more frequent staff visits in the patient rooms and encouraging patients to take part in activities in communal spaces. Finally, we propose that healthcare professionals be involved to a greater extent in the work of creating well-functioning environments for their users.

## References

[cit0001] Anaker, A., Von Koch, L., Heylighen, A., & Elf, M. (2019). ”It’s lonely”: Patients’ experiences of the physical environment at a newly built stroke unit. *HERD: Health Environments Research & Design Journal*, 12(3), 141–14. 10.1177/1937586718806696 30336696 PMC6637812

[cit0002] Anaker, A., Von Koch, L., Sjöstrand, C., Bernhardt, J., & Elf, M. (2017). A comparative study of patients’ activities and interactions in a stroke unit before and after reconstruction—The significance of the built environment. *PLoS One*, 12(7), e0177477. 10.1371/journal.pone.0177477 28727727 PMC5519004

[cit0003] Anaker, A., Von Koch, L., Sjöstrand, C., Heylighen, A., & Elf, M. (2018). The physical environment and patients’ activities and care: A comparative case study at three newly built stroke units. *Journal of Advanced Nursing*, *74*(8), 1919–1931. 10.1111/jan.13690 29676493

[cit0004] Astrand, A., Saxin, C., Sjoholm, A., Skarin, M., Linden, T., Stoker, A., Roshandel, S., Dedering, Å., Halvorsen, M., Bernhardt, J., & Cumming, T. (2016). Poststroke physical activity levels no higher in rehabilitation than in the acute hospital. *Journal of Stroke and Cerebrovascular Diseases*, *25*(4), 938–945. 10.1016/j.jstrokecerebrovasdis.2015.12.046 26851969

[cit0005] Berelson, B. (1952). *Content analysis in communication research*. The Free Press.

[cit0006] Bernhardt, J., Dewey, H., Thrift, A., & Donnan, G. (2004). Inactive and alone: Physical activity within the first 14 days of acute stroke unit care. *Stroke*, *35*(4), 1005–1009. 10.1161/01.STR.0000120727.40792.40 14988574

[cit0007] Blennerhassett, J. M., Borschmann, K. N., Lipson-Smith, R. A., & Bernhardt, J. (2018). Behavioral mapping of patient activity to explore the built environment during rehabilitation. *HERD: Health Environments Research & Design Journal*, *11*(3), 109-123. 10.1177/1937586718758444 29564923

[cit0008] Boulanger, J. M., Lindsay, M. P., Gubitz, G., Smith, E. E., Stotts, G., Foley, N., & Butcher, K. (2018). Canadian stroke best practice recommendations for acute stroke management: Prehospital, emergency department, and acute inpatient stroke care, 6th edition, update 2018. *International Journal of Stroke*, 13(9), 949–984. 10.1177/1747493018786616 Epub 2018 Jul 18 30021503

[cit0009] Brambilla, A., Rebecchi, A., & Capolongo, S. (2019). Evidence based hospital design. A literature review of the recent publications about the EBD impact of built environment on hospital occupants’ and organizational outcomes. *Annali Di Igiene*, 31(2), 165–180. 10.7416/ai.2019.2269 30714614

[cit0010] Chaudhury, H., Hung, L., & Badger, M. (2013). The role of physical environment in supporting person-centered dining in long-term care: A review of the literature. *American Journal of Alzheimer’s Disease & Other Dementias*, 28(5), 491–500. 10.1177/1533317513488923 PMC10852766 23687182

[cit0011] Chaudhury, H., Mahmood, A., & Valente, M. (2009). The effect of environmental design on reducing nursing errors and increasing efficiency in acute care settings a review and analysis of the literature. *Environment and Behavior*, 41(6), 755–786. 10.1177/0013916508330392

[cit0012] Chun, H. Y., Whiteley, W. N., Dennis, M. S., Mead, G. E., & Carson, A. J. (2018). Anxiety after stroke: The importance of subtyping. *Stroke*, 49(3), 556–564. 10.1161/strokeaha.117.020078 29437982 PMC5839706

[cit0013] Clarke, D. (2010). Achieving teamwork in stroke units: The contribution of opportunistic dialogue. *Journal of Interprofessional Care*, 24(3), 285–297. 10.3109/13561820903163645 19995268

[cit0014] Crowfoot, G., Van Der Riet, P., & Maguire, J. (2018). Real-life experiences of people with transient ischaemic attack or minor stroke: A qualitative literature review. *Journal of Clinical Nursing*, 27(7–8), 1381–1398. 10.1111/jocn.14271 29569286

[cit0015] Donetto, S., Penfold, C., Anderson, J., Robert, G., & Maben, J. (2017). Nursing work and sensory experiences of hospital design: A before and after qualitative study following a move to all-single room inpatient accommodation. *Health and Place*, 46, 121–129. 10.1016/j.healthplace.2017.05.001 28527327 PMC5533937

[cit0016] Edvardsson, D., Winblad, B., & Sandman, P. O. (2008). Person-centred care of people with severe alzheimer’s disease: Current status and ways forward. *The Lancet Neurology*, 7(4), 362–367. 10.1016/S1474-4422(08)70063-2 18339351

[cit0017] Elf, M., Frost, P., Lindahl, G., & Wijk, H. (2015). Shared decision making in designing new healthcare environments-time to begin improving quality. *BMC Health Services Research*, *15*(114). 10.1186/s12913-015-0782-7 25888922 PMC4373305

[cit0018] Elo, S., Kääriäinen, M., Kanste, O., Pölkki, T., Utriainen, K., & Kyngäs, H. (2014). Qualitative content analysis: A focus on trustworthiness. *HERD: Health Environments Research & Design Journal*, *4*(1), 1–10. 10.1177/2158244014522633

[cit0019] Elo, S., & Kyngäs, H. (2008). The qualitative content analysis process. *Journal of Advanced Nursing*, 62(1), 107–115. 10.1111/j.1365-2648.2007.04569.x 18352969

[cit0020] Gharaveis, A., Hamilton, D. K., & Pati, D. (2017). The impact of environmental design on teamwork and communication in healthcare facilities: A systematic review. *HERD: Health Environments Research & Design Journal*, *11*(1), 1–19. 10.1177/1937586717730333 29022368

[cit0021] Graneheim, U. H., & Lundman, B. (2004). Qualitative content analysis in nursing research: Concepts,proceduresand measure to achieve trustworthiness. *Nurse Education Today*, 24(2), 105–112. 10.1016/j.nedt.2003.10.001 14769454

[cit0022] Grondin, J. (1995). *Sources of Hermeneutics*. State University of New York Press.

[cit0023] Hackett, M. L., Yapa, C., Parag, V., & Anderson, C. S. (2005). Frequency of depression after stroke: A systematic review of observational studies. *Stroke*, 36(6), 1330–1340. 10.1161/01.STR.0000165928.19135.35 15879342

[cit0024] Hamilton, D. K., & Watkins, D. H. (2009). *Evidence-based design for multiple building types*. John Wiley & Sons.

[cit0025] Harris, P. B., McBride, G., Ross, C., & Curtis, L. (2002). A place to heal: Environmental sources of satisfaction among hospital patients. *Journal of Applied Social Psychology*, 32(6), 1276–1299. 10.1111/j.1559-1816.2002.tb01436.x

[cit0026] Henriksen, K., Isaacson, S., Sadler, L. B., & Zimring, M. C. (2007). The role of the physical environment in crossing the quality chasm. *The Joint Commission Journal on Quality and Patient Safety*, 33(Suppl 11), 68–80. 10.1016/S1553-7250(07)33114-0 18173167

[cit0027] Huisman, E. R. C. M., Morales, E., Van Hoof, J., & Kort, H. S. M. (2012). Healing environment: A review of the impact of built environmental factors on users. *Building and Environment*, 58, 70–80. 10.1016/j.buildenv.2012.06.016

[cit0028] Hung, L., Chaudhury, H., & Rust, T. (2016). The effect of dining room physical environmental renovations on person-centered care practice and residents’ dining experiences in long-term care facilities. *Journal of Applied Gerontology*, 35(12), 1279–1301. 10.1177/0733464815574094 25724947

[cit0029] Janssen, H., Ada, L., Bernhardt, J., McElduff, P., Pollack, M., Nilsson, M., & Spratt, N. J. (2014). An enriched environment increases activity in stroke patients undergoing rehabilitation in a mixed rehabilitation unit: A pilot non-randomized controlled trial. *Disability and Rehabilitation*, 36(3), 255–262. 10.3109/09638288.2013.788218 23627534

[cit0030] Joseph, A., Choi, Y. S., & Quan, X. (2015). Impact of the physical environment of residential health, care, and support facilities (RHCSF) on staff and residents: A systematic review of the literature. *Environment and Behavior*, *48*(10), 1203-1241. 10.1177/0013916515597027

[cit0031] Kilner, E., & Sheppard, L. A. (2010). The role of teamwork and communication in the emergency department: A systematic review. *International Emergency Nursing*, 1(8), 127–137. 10.1016/j.ienj.2009.05.006 20542238

[cit0032] Kirkevold, M. (2010). The role of nursing in the rehabilitation of stroke survivors: An extended theoretical account. *Advances in Nursing Science*, 33(1), E27–E40. 10.1097/ANS.0b013e3181cd837f 20154522

[cit0033] Krippendorff, K. (2004). *Content analysis. An introduction to its methodology* (2nd ed.). Sage Publications.

[cit0034] Krippendorff, K. (2013). *Content analysis: An introduction to its methodology*. Sage.

[cit0035] Kuys, S. S., Dolecka, U. E., & Morrison, C. A. (2011). Appropriate seating for medical patients: An audit. *Australian Health Review*, *35*(3), 316–319. 10.1071/AH10943 21871193

[cit0036] Langhorne, P., & Pollock, A. (2002). What are the components of effective stroke unit care? *Age Ageing*, 31(5), 365–371. 10.1093/ageing/31.5.365 12242199

[cit0037] Leaman, A., Stevenson, F., & Bordass, B. (2010). Building evaluation: Practice and principles. *Building Research & Information*, 38(5), 564–577. 10.1080/09613218.2010.495217

[cit0038] Lincoln, Y., & Guba, E. G. (1985). *Naturalistic inquiry*. Sage.

[cit0039] Maben, J., Griffiths, P., Penfold, C., Somon, M., Anderson, J. E., Robert, G., & Barlow, J. (2016). One size fits all? Mixed methods evaluation of the impact of 100% single-room accommodation on staff and patient experience, safety and costs. *BMJ Quality & Safety*, *25*(4), 241–256. 10.1136/bmjqs-2015-004265. PMC4819646 26408568

[cit0040] Malenbaum, S., Keefe, F. J., Williams, A., Ulrich, R., & Somers, T. J. (2008). Pain in its environmental context: Implications for designing environments to enhance pain control. *Pain*, 134(3), 241–244. 10.1016/j.pain.2007.12.002 18178010 PMC2264925

[cit0041] McCormack, B., McCormack, B., & McCance, T. (2010). *Person-centred nursing [electronic resource]: Theory and practice*. John Wiley & Sons Ltd. 10.1002/9781444390506

[cit0042] Miller, E. L., Murray, L., Richards, L., Zorowitz, R. D., Bakas, T., Clark, P., & Billinger, S. A. (2010). Comprehensive overview of nursing and interdisciplinary rehabilitation care of the stroke patient: A scientific statement from the American Heart Association. *Stroke*, 41(10), 2402–2448. 10.1161/STR.0b013e3181e7512b 20813995

[cit0043] Morgan-Brown, M., Newton, R., & Ormerod, M. (2013). Engaging life in two Irish nursing home units for people with dementia: Quantitative comparisons before and after implementing household environments. *Aging & Mental Health*, 17(1), 57–65. 10.1080/13607863.2012.717250 22943667

[cit0044] Nordin, S., McKee, K., Wijk, H., & Elf, M. (2017). The association between the physical environment and the well-being of older people in residential care facilities: A multilevel analysis. *Journal of Advanced Nursing*, 73(12), 2942–2952. 10.1111/jan.13358 28586513

[cit0045] O’Halloran, R., Grohn, B., & Worrall, L. (2012). Environmental factors that influence communication for patients with a communication disability in acute hospital stroke units: A qualitative metasynthesis. *Archives of Physical Medicine & Rehabilitation*, 93(1 Suppl), S77–85. 10.1016/j.apmr.2011.06.039 22119075

[cit0046] Pati, D., Pati, S., & Harvey, T. E. (2016). Security implications of physical design attributes in the emergency department. *HERD: Health Environments Research & Design Journal*, 9(4), 50–63. 10.1177/1937586715626549 26794235

[cit0047] Persson, E., & Määttä, S. (2012). To provide care and be cared for in a multiple-bed hospital room. *Scandinavian Journal Of Caring Sciences*, 26(4), 663–670. 10.1111/j.1471-6712.2012.00976.x 22390650

[cit0048] Polit, D. F., & Beck, C. T. (2012). *Nursing research: Principles and methods*. Lippincott Williams & Wilkins.

[cit0049] Powers, W. J., Rabinstein, A. A., Ackerson, T., Adeoye, O. M., Bambakidis, N. C., Becker, K., & Tirschwell, D. L. (2019). Guidelines for the early management of patients with acute ischemic stroke: 2019 update to the 2018 guidelines for the early management of acute ischemic stroke: A guideline for healthcare professionals from the American Heart Association/American Stroke Association. *Stroke*, 50(12), e344–e418. 10.1161/STR.0000000000000211 Epub 2019 Oct 30 31662037

[cit0050] Ringelstein, E. B., Chamorro, A., Kaste, M., Langhorne, P., Leys, D., Lyrer, P. …., & Toni, D. (2013). European stroke Organisation recommendations to establish a stroke unit and stroke center. *Stroke*, 44(3), 828–840. 10.1161/strokeaha.112.670430 23362084

[cit0051] Rosbergen, I. C., Grimley, R. S., Hayward, K. S., & Brauer, S. G. (2019). The impact of environmental enrichment in an acute stroke unit on how and when patients undertake activities. *Clinical Rehabilitation*, 33(4), 784–795. 10.1177/0269215518820087 30582368

[cit0052] Rosbergen, I. C., Grimley, R. S., Hayward, K. S., Walker, K. C., Rowley, D., Campbell, A. M., Brauer, S. G., McGufficke, S., Robertson, S. T., Trinder, J., & Janssen, H. (2017). Embedding an enriched environment in an acute stroke unit increases activity in people with stroke: A controlled before-after pilot study. *Clinical Rehabilitation*, 31(11), 1516–1528. 10.1177/0269215517705181 28459184

[cit0053] Seneviratne, C. C., Mather, C. M., & Then, K. L. (2009). Understanding nursing on an acute stroke unit: Perceptions of space, time and interprofessional practice. *Journal of Advanced Nursing*, 65(9), 1872–1881. 10.1111/j.1365-2648.2009.05053.x 19694850

[cit0054] Shannon, M. M., Elf, M., Churilov, L., Olver, J., Pert, A., & Bernhardt, J. (2018). Can the built environment itself influence neurological patient activity? *Disability and Rehabilitation*, *41*(10), 1177-1189. 10.1080/09638288.2017.1423520 29343110

[cit0055] Stankos, M., & Schwarz, B. (2007). Evidence-based design in healthcare: A theoretical dilemma. *Interdisciplinary Design and Research e-Journal*, 1(1).

[cit0056] Stroke Foundation. (2017) . *Clinical guidelines for stroke management 2017*. Stroke Foundation Melbourne, Victoria, Australiaguidelines@strokefoundation.org.au

[cit0057] Stroke Unit Trialists’ Collaboration. (2013). Organised inpatient (stroke unit) care for stroke. *Cochrane Database of Systematic Reviews*, 9, CD000197. 10.1002/14651858.CD000197.pub3 PMC6474318 24026639

[cit0058] Taylor, E., & Hignett, S. (2016). The SCOPE of hospital falls: A systematic mixed studies review. *HERD: Health Environments Research & Design Journal*, 9(4), 86–109. 10.1177/1937586716645918 27240563

[cit0059] Taylor, E., McKevitt, C., & Jones, F. (2015). Factors shaping the delivery of acute inpatient stroke therapy: A narrative synthesis. *Journal of Rehabilitation Medicine*, 47(2), 107–119. 10.2340/16501977-1918 25437308

[cit0060] Tong, A., Sainsbury, P., & Craig, J. (2007). Consolidated criteria for reporting qualitative research (COREQ): A 32-item checklist for interviews and focus groups. *International Journal for Quality in Health Care*, 19(6), 349–357. 10.1093/intqhc/mzm042 17872937

[cit0061] Tough, H., Siegrist, J., & Fekete, C. (2017). Social relationships, mental health and wellbeing in physical disability: A systematic review. *BMC Public Health*, 17(414), 1-18. 10.1186/s12889-017-4308-6 PMC5422915 28482878

[cit0062] Towfighi, A., Ovbiagele, B., El Husseini, N., Hackett, M. L., Jorge, R. E., Kissela, B. M., Williams, L. S., Mitchell, P. H., Skolarus, L. E., & Whooley, M. A. (2017). Poststroke depression: A scientific statement for healthcare professionals from the American Heart Association/American Stroke Association. *Stroke*, 48(2), e30–e43. 10.1161/str.0000000000000113 27932603

[cit0063] Ulrich, R. S., Berry, L. L., Quan, X., & Parish, J. T. (2010). A conceptual framework for the domain of evidence-based design. *HERD: Health Environments Research & Design Journal*, 24 (1), 95–114. Epub 2010/ 12/18. PMID: 21162431. 10.1177/193758671000400107 21162431

[cit0064] Ulrich, R. S., Zimring, C., Quan, X., & Joseph, A. (2006). The environment’s impact on stress. In S. Marberry (Ed.), *Improving healthcare with better building design* (pp. 37–61). Health Administration Press.

[cit0065] Ulrich, R. S., Zimring, C., Zhu, X., DuBose, J., Seo, H. B., Choi, Y. S., & Joseph, A. (2008). A review of the research literature on evidence-based healthcare design. *HERD: Health Environments Research & Design Journal*, 1(3), 61–125. 10.1177/193758670800100306 21161908

[cit0066] Villa, R. F., Ferrari, F., & Moretti, A. (2018). Post-stroke depression: Mechanisms and pharmacological treatment. *Pharmacology & Therapeutics*, 184, 131–144. 10.1016/j.pharmthera.2017.11.005 29128343

[cit0067] Vischer, J. C. (2008). Towards a user-centred theory of the built environment. *Building Research & Information*, 36(3), 231–240. 10.1080/09613210801936472

[cit0068] White, J. H., Alborough, K., Janssen, H., Spratt, N., Jordan, L., & Pollack, M. (2014). Exploring staff experience of an “enriched environment” within stroke rehabilitation: A qualitative sub-study. *Disability & Rehabilitation*, 36(21), 1783–1789. 10.3109/09638288.2013.872200 24369101

[cit0069] World Health Organization. (2004) . *Global burden of stroke. The atlas of heart disease and stroke*.

[cit0070] World Medical Association. (2013). World medical association declaration of Helsinki: Ethical principles for medical research involving human subjects. *Jama*, 310(20), 2191. 10.1001/jama.2013.281053 24141714

[cit0071] Zamani, Z. (2019). Effects of emergency department physical design elements on security, wayfinding, visibility, privacy, and efficiency and its implications on staff satisfaction and performance. *HERD: Health Environments Research & Design Journal*, 12(3), 72–88. 10.1177/1937586718800482 30231637

[cit0072] Zeisel, J. (2013). Improving person-centered care through effective design. *Generations*, 37(3), 45–52. https://www.jstor.org/stable/26591680

